# Impaired Left Ventricular Contractile Reserve in Patients With Hypertrophic Cardiomyopathy and Abnormal Blood Pressure Response: A Stress Echocardiographic Study

**DOI:** 10.7759/cureus.32145

**Published:** 2022-12-02

**Authors:** Mayumi Takeoka, Michiyo Yamano, Sakiko Honda, Chieko Sakai, Tatsuya Kawasaki

**Affiliations:** 1 Department of Cardiology, Matsushita Memorial Hospital, Moriguchi, JPN; 2 Department of Cardiovascular Medicine, Kyoto Prefectural University of Medicine, Kyoto, JPN

**Keywords:** doppler echocariography, 2d-speckle tracking, exercise stress echocardiography, abnormal blood pressure response, hypertrophic cardiomyopathy

## Abstract

Background: Abnormal blood pressure response (ABPR) has been reported to be a risk factor for sudden cardiac death in patients with hypertrophic cardiomyopathy (HCM). We aimed to elucidate the relationship between ABPR during exercise stress echocardiography (ESE) and impaired left ventricular (LV) contractile reserve based on two-dimensional strain in patients with HCM.

Methods: Patients with HCM underwent ESE with treadmill exercise. Patients whose blood pressure elevation at maximum workload was lower than 20 mmHg from baseline were classified as having ABPR. Echocardiographic parameters were compared between patients with and without ABPR.

Results: Of 26 patients with HCM, nine patients were diagnosed with ABPR. Significant LV outflow tract obstruction (>50 mmHg) was provoked only in one patient with ABPR (baseline to the conclusion of the exercise, 15.2 mmHg to 63.0 mmHg). Change in cardiac output (CO) and the ratio of early diastolic velocity to early annular velocity (E/e') from baseline to just after the conclusion of exercise did not differ between patients with and without ABPR (CO, 102±40% vs. 122±45%, *P* = 0.19; E/e', 4±22% vs. 2±20%, *P* = 0.86). Change in systemic vascular resistance change was not significant (patients with vs. without ABPR, -52±10% vs. -46±13%, *P* = 0.24). Percent change in LV global longitudinal strain was lower in patients with ABPR than patients without ABPR (12±17% vs. 27±15%, *P* = 0.02).

Conclusion: In conclusion, impaired LV contractile reserve during exercise might contribute to ABPR in patients with HCM.

## Introduction

Abnormal blood pressure response (ABPR) has been reported to be a risk factor for sudden cardiac death in patients with hypertrophic cardiomyopathy (HCM) [1]. Although a mechanism responsible for ABPR has been proposed by some authors [2-4], there is no established explanation for this phenomenon. HCM is generally known to be preserved its contractility based on visual echocardiographic assessment, but there have been patients whose left ventricular (LV) strain had decreased [5]. Impairment of percentage change in systolic strain rate post-exercise has been associated with poor exercise capacity or new-onset ventricular tachycardia [6]. We aimed to elucidate the relationship between ABPR during exercise stress echocardiography (ESE) and impaired LV strain in patients with HCM.

## Materials and methods

Study subjects

Thirty-one consecutive patients with HCM underwent ESE during treadmill exercise. HCM was diagnosed based on maximum wall thickness ≥15 mm calculated by transthoracic echocardiography in the absence of any cardiac or systemic disorder that could cause hypertrophy [7]. Patients whose intraventricular peak pressure gradient was ≥30 mmHg at rest were not included in this study [7]. Patients with optimal medical therapy for pathological intraventricular obstruction were also excluded. Five patients were excluded: one had archived echocardiographic images that were inadequate for analyzing the parameters, two had undergone surgical myectomy, and two had atrial fibrillation. Finally, 26 patients were included in the analysis of ABPR. Patients with epicardial coronary stenosis were not included in this study. All study patients gave written informed consent. The study was approved by the ethics committee of Matsushita Memorial Hospital (ERB-21029).

ESE

All patients underwent treadmill ESE with target heart rate as the goal using the Bruce protocol or the modified Bruce protocol. Patients were asked to continue taking beta-blockers or calcium antagonists on the day of ESE. We used the modified Bruce protocol when a patient’s exercise tolerance was predicted to be <4 metabolic equivalents (METs) based on the interview before the ESE [8,9]. We also calculated the ratio of METs achieved to age-based and gender-based predictions [10,11]. Blood pressure, 12-lead electrocardiography, and pulse oximetry were monitored continuously during and up to 5 minutes after the conclusion of the exercise. ABPR was defined as a decrease in systolic blood pressure or an increase in blood pressure lower than 20 mmHg [2,7,12]. Patients were divided into two groups: patients with ABPR and without ABPR.

Conventional echocardiographic parameters, including LV diastolic parameters, were assessed before the exercise test and just after the conclusion of the exercise (E9, GE Healthcare, Milwaukee, WI, USA). LV volumes and ejection fraction were assessed using the modified Simpson’s method based on apical four-chamber and two-chamber views. Cardiac output was determined based on LV stroke volume, which was calculated by multiplying LV outflow tract area by LV outflow tract velocity-time integral measured with pulsed Doppler. We also calculated LV global longitudinal strain (GLS) based on apical four-chamber, two-chamber, and long-axis views [13]. Patients were defined as having exercise-induced pulmonary hypertension if the peak tricuspid regurgitation pressure gradient after the conclusion of the exercise was >50 mmHg [14,15]. We analyzed systemic vascular resistance using the following formula: (mean arterial pressure - right atrial pressure)/cardiac output × 80 dynes/sec/cm^5^ [16]. Echocardiographic parameters at baseline and just after the conclusion of the exercise were compared between patients with and without ABPR.

Subendocardial ischemia

Subendocardial ischemia was assessed with technetium-99m tetrofosmin myocardial scintigraphy, as previously described [17,18]. The left ventricle was divided into 15 short-axis slices, and 100 radii were generated at 3.6-degree intervals from the center of the left ventricle in each image. An area surrounded by 100 points, each representing the maximum count on a radius, was calculated in stress and post-exercise images. Subendocardial ischemia was considered positive if the stress to the post-exercise ratio in 15 surface areas is greater than the mean plus 2 standard deviations in normal subjects [19]. Scintigraphy was performed to diagnose subendocardial ischemia in 22 of 26 patients.

Statistical analysis

All normally distributed values are expressed as means ± standard deviation. The characteristics of patients with and without ABPR were compared with the chi-square test for categorical variables and unpaired Student’s t-tests for continuous variables. Changes (∆) in echocardiographic parameters from baseline to the conclusion of exercise were calculated as follows: ∆ / value at baseline (%). A two-sided P-value <0.05 was considered statistically significant. All statistical analyses were performed using IBM Corp. Released 2020. IBM SPSS Statistics for Windows, Version 27.0. Armonk, NY: IBM Corp. 

## Results

**Figure 1:**
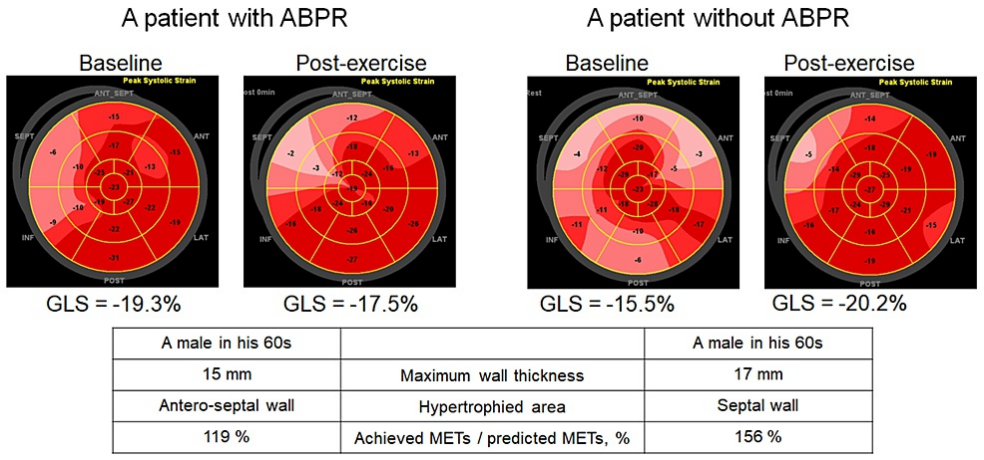
Representative cases of ABPR and no ABPR Although the exercise load was enough in both the patients, they had a different change in LV GLS.

LV hypertrophy was observed in the following areas: anteroseptal wall (n=5), septum (n=9), septum and free wall (n=4), and other (n=8). Baseline clinical and echocardiographic parameters are shown in Table 1. Table 2 shows exercise test values. Exercise tests were terminated due to electrocardiographic changes in three patients: one patient had ST-T depression ≥2.0 mm, and two had non-sustained ventricular tachycardia. Stroke volume augmentation was observed in both groups (21±12% in patients with ABPR and 14±19% in patients without ABPR) (Table 3). Furthermore, a significant increase in cardiac output was observed in both groups (102±40% in patients with ABPR and 122±45% in patients without ABPR). LV outflow tract obstruction >50 mmHg was provoked in one patient with ABPR (baseline to the conclusion of the exercise, 15.2 mmHg to 63.0 mmHg). LV GLS improved after the conclusion of the exercise in patients without ABPR but not in patients with ABPR (27±15% in patients without ABPR and 12±17% in patients with ABPR, P = 0.02) (Table 3). Representative cases of ABPR and no ABPR are shown in Figure 1. Regarding LV diastolic parameters, the ratio of early diastolic velocity (E) to septal early diastolic annular velocity (e') did not change significantly from baseline to the conclusion of the exercise in both groups. Change in systemic vascular resistance did not differ between the two groups (patients with vs. without ABPR, -52±10% vs. -46±13%, P = 0.24). Exercise-induced pulmonary hypertension was observed in only one patient without ABPR.

**Table 1:** Baseline characteristics ABPR: Abnormal blood pressure response; ACE: Angiotensin-converting enzyme; ARB: Angiotensin II receptor blocker; HCM: Hypertrophic cardiomyopathy; LV: Left ventricular; NYHA: New York Heart Association.

	ABPR (+) (n = 9)	ABPR (-) (n = 17)	P value
Age, years	67 ± 7	62 ± 11	0.19
Male/female	7 / 2	15 / 2	0.48
Body mass index, kg/m^2^	25.3 ± 2.0	23.9 ± 2.5	0.16
NYHA functional class, Ⅰ/Ⅱ/Ⅲ/Ⅳ	8 / 1 / 0 / 0	15 / 2 / 0 / 0	0.96
Family history of sudden cardiac death, n (%)	1 (11)	2 (12)	0.96
Family history of HCM, n (%)	3 (33)	13 (76)	0.03
History of syncope, n (%)	0	0	-
Beta-blocker, n (%)	1 (11)	2 (12)	0.96
Calcium antagonist, n (%)	1 (11)	4 (24)	0.45
ACE inhibitor/ARB, n (%)	3 (33)	2 (12)	0.18
Maximum LV wall thickness, mm	16 ± 2	16 ± 2	0.69
LV end-diastolic diameter, mm	42 ± 4	43 ± 6	0.63
Left atrial volume index, mL/m^2^	42 ± 8	43 ± 10	0.68

**Table 2:** Exercise test values in patients with and without ABPR ABPR: Abnormal blood pressure response; BP: Blood pressure; ECG: Electrocardiography; METs: Metabolic equivalents.

	ABPR (+)	ABPR (-)	P value
Exercise time, min	9.5 ± 3.8	10.0 ± 2.9	0.72
Target heart rate, bpm	130 ± 6	134 ± 9	0.19
Heart rate at maximum exercise, bpm	128 ± 13	138 ± 15	0.13
Heart rate at maximum exercise/target heart rate, %	98 ± 8	102 ± 8	0.25
Systolic BP at maximum exercise, mmHg	146 ± 13	181 ± 24	< 0.01
Diastolic BP at maximum exercise, mmHg	74 ± 13	79 ± 18	0.55
Change in systolic BP from baseline, mmHg	-3.6 ± 15.6	46.5 ± 18.1	< 0.01
Change in diastolic BP from baseline, mmHg	-7.1 ± 23.5	-1.6 ± 16.6	0.49
METs achieved	6.8 ± 2.8	7.7 ± 2.7	0.42
METs achieved/METs predicted, %	113 ± 42	116 ± 33	0.85
Cause of exercise cessation			0.32
Attainment of target heart rate, n (%)	6 (67)	11 (65)	
Dyspnea, n (%)	0	3 (18)	
Significant ECG change, n (%)	1 (11)	2 (12)	
Leg fatigue, n (%)	1 (11)	0	
Oxygen desaturation, n (%)	1 (11)	0	
Excessive BP increase, n (%)	0	1 (6)	

**Table 3:** Echocardiographic parameters at baseline and just after the conclusion of exercise DT: Deceleration time of transmitral early filling flow; E: Early diastolic velocity; E/A: Ratio of E to A (late diastolic velocity); e': Early diastolic annular velocity; LV: Left ventricular; MR: Mitral regurgitation; TRPG: Peak tricuspid regurgitation pressure gradient. *P <0.05 vs. baseline value in patients with ABPR; †P <0.05 vs. post-exercise value in patients with ABPR; ‡P <0.05 vs. patients with ABPR.

	ABPR (+)			ABPR (-)		
	Baseline	Post-exercise	% change	Baseline	Post-exercise	% change
LV end-diastolic volume, mL	78 ± 25	89 ± 30	15 ± 21	81 ± 22	86 ± 23	8 ± 17
LV end-systolic volume, mL	33 ± 13	34 ± 16	5 ± 38	32 ± 12	31 ± 13	-2 ± 22
LV stroke volume, mL	45 ± 13	55 ± 17	21 ± 12	49 ± 11	55 ± 11	14 ± 19
LV ejection fraction, %	58 ± 5	62 ± 9	8 ± 13	62 ± 6	65 ± 6	7 ± 10
Cardiac output, L/min	3.5 ± 1.2	7.1 ± 2.5	102 ± 40	3.4 ± 0.7	7.5 ± 1.9	122 ± 45
LV GLS, %	-14.6 ± 3.4	-15.9 ± 3.5	12 ± 17	-15.4 ± 3.6	-19.4 ± 4.0^†^	27 ± 15^‡^
E, m/s	0.63 ± 0.20	0.81 ± 0.20	33 ± 34	0.66 ± 0.13	0.94 ± 0.17	43 ± 20
E/A	0.97 ± 0.66	1.30 ± 1.06	27 ± 32	1.13 ± 0.45	1.22 ± 0.48	16 ± 43
DT, ms	253 ± 199	156 ± 72	-36 ± 22	179 ± 41^*^	146 ± 68	-17 ± 32
Septal e', cm/s	4.0 ± 0.8	5.2 ± 1.4	31 ± 32	4.5 ± 1.1	6.4 ± 1.5	43 ± 26
Septal E/e'	16.2 ± 6.0	16.7 ± 6.4	4 ± 22	15.4 ± 5.1	15.4 ± 4.5	2 ± 20
Peak TRPG, mmHg	16.9 ± 5.0	23.6 ± 8.2	43 ± 46	19.1 ± 6.1	31.2 ± 14.9	63 ± 52
MR grade none/I/II, n	2/7/0	1/8/0	-	2/15/0	2/14/1	-

Subendocardial ischemia was detected in eight of 22 patients who underwent scintigraphy. The incidence of subendocardial ischemia was higher in patients with ABPR (43% in patients with ABPR vs. 33% in patients without ABPR, P <0.01). Improvement in LV GLS after ESE was similar between patients with and without subendocardial ischemia (22 ± 18% vs. 21 ± 18%, P = 0.89).

## Discussion

The main finding of our study was that LV contractile reserve estimated based on LV GLS during ESE was impaired in patients with HCM and ABPR, even though changes in cardiac output and systemic vascular resistance from baseline to just after the conclusion of exercise were similar in patients with and without ABPR.

ABPR in patients with HCM

The incidence of ABPR has been reported to be higher in younger patients, patients with a small LV cavity, and patients with a family history of cardiac sudden death [2]. Although there was no definitive explanation, possible mechanisms for ABPR have been proposed. Impaired LV filling due to tachycardia during exercise was considered a cause of ABPR. Frenneaux et al. reported changes in the cardiac index based on Swan-Ganz catheter measurements during treadmill exercise in HCM patients with ABPR and without ABPR [2]. The cardiac index increased five-fold in both groups at peak exercise, but it was significantly higher in patients with ABPR. Systemic vascular resistance in patients with ABPR was significantly lower than in patients without ABPR. On the other hand, Nagata et al. performed a radionuclide monitoring study during supine ergometer exercise in HCM patients with and without ABPR [3]. Although systemic vascular resistance was similar in the two groups, changes in LV stroke volume from baseline to maximum exercise were significantly smaller in patients with ABPR than in those without ABPR in their study. In our study, neither change in LV stroke volume nor change in systemic vascular resistance differed between patients with and without ABPR. 

LV GLS in HCM

Parameters based on speckle tracking were reported to be useful and important in the characterization and prediction of poor prognosis in patients with HCM and normal LV ejection fraction. Carasso et al. analyzed differences in longitudinal strain, circumferential strain, and a combination of these strains in patients with HCM and controls [5]. The circumferential strain was higher and longitudinal strain was significantly impaired in patients with HCM versus normal controls despite similar LV ejection fraction. Other authors reported impaired LV longitudinal strain in patients with HCM and preserved LV ejection [20]. Changes in strain parameters during ESE have been rarely published. In a study of patients with hypertensive hypertrophy, patients with HCM and normal controls underwent ESE, change in peak longitudinal strain was calculated from apical four-chamber and two-chamber views from baseline to just after the conclusion of treadmill exercise; the circumferential strain was also analyzed [21]. Changes in longitudinal strain and strain rate were significantly lower in patients with HCM. Thus, there was a significant direct relationship between rate-pressure product and change in strain in that study, but differences among patients with HCM were not evaluated. Pozios et al. also reported changes in strain and strain rate during ESE in patients with HCM [6]. Changes in strain rate and post-exercise strain rate were significantly correlated with exercise capacity and the extent of late gadolinium enhancement; they were predictors of ventricular tachycardia. 

Subendocardial ischemia and change in strain

In the assessment of exercise-induced abnormalities in patients with HCM, transient LV cavity dilatation without coronary stenosis, i.e., subendocardial ischemia, has been often reported [19,22]. Subendocardial ischemia had been reported to be associated with elevated LV filling pressure [23]. Evidence of subendocardial ischemia from echocardiography has been lacking. Previous studies that examined wall motion abnormalities or impaired strain during ESE [6,21,24] might be associated with subendocardial ischemia. Vagal nerves are predominantly distributed in the LV subendocardium based on published results of histological examinations and an experimental study [25,26]. We previously reported that subendocardial ischemia is associated with the enhancement of vagal modulation and might be related to ABPR in patients with HCM [4]. Thus, impaired LV GLS after exercise observed in patients with subendocardial ischemia seems reasonable. Impairment of changes in LV GLS with exercise might be related to subendocardial ischemia. In addition, LV contractile reserve might be related to ABPR. The incidence of subendocardial ischemia diagnosed with scintigraphy was significantly higher in patients with ABPR than without ABPR in our study. However, patients with subendocardial ischemia did not coincide completely with patients with ABPR. One considerable reason is as follows: LV GLS is calculated as the mean from all layers from the endocardial side to the epicardial side by the software we used in this study. Therefore, it might be difficult to detect impairments in strain on the endocardial side. Further validation studies will be needed to examine the findings of subendocardial ischemia with ESE and scintigraphy.

Limitations

First, this was a single-center retrospective cohort analysis. Second, we performed an echocardiographic evaluation of hemodynamic status. Since we did not measure cardiac output and LV diastolic pressure with cardiac catheterization, there might have been errors in estimating these parameters. However, we use currently accepted guidelines for estimating hemodynamic status with echocardiographic parameters. Third, there was a variation in the duration between ESE and scintigraphy due to the retrospective nature of this assessment.

## Conclusions

ABPR during ESE using treadmill test was observed in nine out of 26 patients with HCM. HCM patients with ABPR had impaired change in LV GLS during ESE when compared with HCM patients without ABPR, though changes in cardiac output and systemic vascular resistance from baseline to just after the conclusion of exercise were similar in both groups. Impaired contractile reserve estimated based on LV GLS might contribute to ABPR in patients with HCM.
